# Muscle Moment Arm–Joint Angle Relations in the Hip, Knee, and Ankle: A Visualization of Datasets

**DOI:** 10.1007/s10439-025-03735-w

**Published:** 2025-05-09

**Authors:** Ziyu Chen, David W. Franklin

**Affiliations:** 1 Neuromuscular Diagnostics, TUM School of Medicine and Health, Technical University of Munich, Munich, Germany; 2 Munich Institute of Robotics and Machine Intelligence (MIRMI), Technical University of Munich, Munich, Germany; 3 Munich Data Science Institute (MDSI), Technical University of Munich, Munich, Germany

**Keywords:** Center of rotation, Joint moment, Moment arm, Muscle force, Tendon excursion

## Abstract

Muscle moment arm is a property that associates muscle force with joint moment and is crucial to biomechanical analysis. In musculoskeletal simulations, the accuracy of moment arm is as important as that of muscle force, and calibrating moment arms in a musculoskeletal model requires data from anatomical measurements. Nonetheless, such data are elusive, and the complex relation between moment arm and joint angle can be unclear. Using common techniques in systematic review, we collected a total of 300 moment arm datasets from literature and visualized the muscle moment arm–joint angle relations in the human hip, knee, and ankle. The findings contribute to the analysis of musculoskeletal mechanics and providing reference regarding the experimental design for future moment arm measurements.

**Supplementary Information:**

The online version contains supplementary material available at 10.1007/s10439-025-03735-w.

## Introduction

The movement of the human body is in essence the compound motion of joints actuated by torques. More precisely, it is driven by *moments of force*, since the actuation originates from forces exerted by muscles, which are not force couples such as what generally arise from electric motors [1, 2]. Thus, an important difference between the studies of robotics and human kinetics is that biomechanists must take into account the property that transforms muscle force into joint moment, namely muscle moment arm [3–5]. The sign of moment arm determines the action of the muscle at a joint, e.g., whether it is an extensor or flexor, and its magnitude partially determines the level of muscle activation required in a motion.

In simple cases, moment arm can be perceived as the distance between the muscle line of action and the center of rotation (CoR), and many experiments have utilized this idea to measure moment arm [6–10]. However, the path of a muscle is often not straight, and the distance between its *curve* of action and the rotation center may not be constant (imagine a cable wrapping around a cam), even when the muscle is isometric and at rest. In such scenario, the CoR method could be inaccurate due to the difficulty of determining the correct perpendicular foot, and one will need to visit the precise definition of moment arm for solutions.

In strict terms, joint moment is associated with muscle force by:

1 $$\begin{aligned} \pmb {\tau } = F\pmb r \end{aligned}$$

where $\pmb \tau$ is an $N\times 1$ vector of moment, *F* is a scalar of force, and $\pmb r$ is an $N\times 1$ vector of moment arm; *N* is the number of degrees of freedom (DoF) in the system.

Hence, if both joint moment and muscle force are known, moment arm is easily calculated as their quotient [11]. However, although joint moment is simple to measure, muscle force is difficult to obtain in vivo, and this concept of *kinetic balance* (KB) is only occasionally employed in cadaveric studies [12, 13].

A practical alternative is to follow the principle of virtual work [11, 14]: If a frictionless system is assumed, then in a sufficiently small period, the work done by the muscle should theoretically equal to the work done on the joint:

2 $$\begin{aligned} \tau d\theta&= F dl \nonumber \\ \tau&= F \frac{dl}{d\theta } \end{aligned}$$

where $d\theta$ and *dl* are small displacements in joint angle and muscle length.

Then, from Eq. (1), we may have:

3 $$\begin{aligned} r = \frac{dl}{d\theta } \end{aligned}$$

which serves as an alternative definition for moment arm. With this, moment arm can be calculated based on the changes in joint angle and muscle length, the latter of which is much easier to measure in vivo than muscle force. This is the tendon excursion (TE) method and frequently used for moment arm measurement [8, 15–18].

One other definition of moment arm surfacing in literature [4, 19, 20] is:

4 $$\begin{aligned} \vec {r} = (\vec {\omega } \cdot \vec {p} \times \vec {F}) \vec {\omega } \end{aligned}$$

where $\vec {\omega }$ denotes the axial direction of joint rotation in a 3D Cartesian space, $\vec {F}$ is a 3D unit vector denoting the direction of the muscle line of action, and $\vec {p}$ is a 3D position vector from any point on the rotation axis to the muscle line of action. Note that $\vec {r}$ is a 3D vector of moment arm, different from $\pmb r$ in Eq. (1); specifically, the 2-norm of $\vec {r}$ could be one of the elements in the vector $\pmb r$.

Critically, this definition assumes the existence of the line of action. Therefore, for application, one must decide which part of the muscle is treated as the straight path to yield $\vec {F}$ for moment arm calculation, and this is essentially still the CoR method.

With the relation between kinetics and moment arm elucidated, it is clear how crucial moment arm is to biomechanical analysis. For example, for accurate musculoskeletal simulation, there has been a major focus on contraction dynamics, such as improving muscle models [21, 22], investigating the sensitivity of muscle force estimation to musculotendon parameters [23–25], and performing large-scale human measurements for model calibration [26, 27]. Nevertheless, these efforts contribute solely to the accuracy of *F* in Eq. (1), while $\pmb r$ is equally crucial in predicting moment output or estimating muscle activation. It is thus important to know how moment arm changes with different joint positions, as well as which muscles lack sufficient measurement of moment arm.

The purpose of this study is to collect existing datasets from literature constituting moment arm–joint angle relations in the hip, knee, and ankle. We approach the issue using common techniques in systematic review and we aim to visualize data with sufficient details to provide reference values for biomechanical analysis, especially musculoskeletal modeling.

## Methods

Moment arm in the human lower limb is investigated by muscle group. For each muscle group, we used a combination of keywords to initiate batch searches in Google Scholar (conducted in November, 2023) with the software Publish or Perish (Version 8) [28]. To guarantee search efficiency, we experimented multiple combinations of keywords in search of 16 target studies with Achilles tendon moment arm data [6, 8, 9, 29–41] and 7 studies with patellar tendon [19, 20, 42–46]. The keyword set selected for the final batch search is one that led to the most target papers while having them appear in top search rankings: For the Achilles tendon, it was 12 out of 16 and on average 11th place in the list of 200 results, while it was 7 out of 7 and 18th place for the patellar tendon.

The format of this keyword set is shown in Table 1, where the number of searches for each muscle group is based on muscle size [26]. We presume it is easier to measure moment arm on large muscles, so there should be more studies reporting relevant data, and more searches are required for larger muscles. In total, 4400 searches were initiated for 21 muscles or muscle groups.

**Table 1:** Number of searches and keywords for each muscle or muscle group

Number of searches	Keyword set (Part 1)^a^
500	(Triceps surae OR achilles)
	(Quadriceps OR patella)
	Gluteus
	(Hamstring OR biceps femoris OR semimembranosus OR semitendinosus)
200	(Iliopsoas OR iliacus OR psoas)
	Tibialis posterior
	Adductor AND (magnus OR brevis OR longus)
	Flexor AND (hallucis OR digitorum longus)
	Peroneus
	Tibialis anterior
	Extensor AND (hallucis OR digitorum longus)
100	Pectineus
	Piriformis
	Gracilis
	Tensor fascia
	Sartorius
	Obturator
	Popliteus
	(Gemelli OR gemellus)
	Quadratus femoris
	Plantaris

^a^Part 2: [...] AND moment arm AND (rotation OR excursion)

**Fig. 1:**
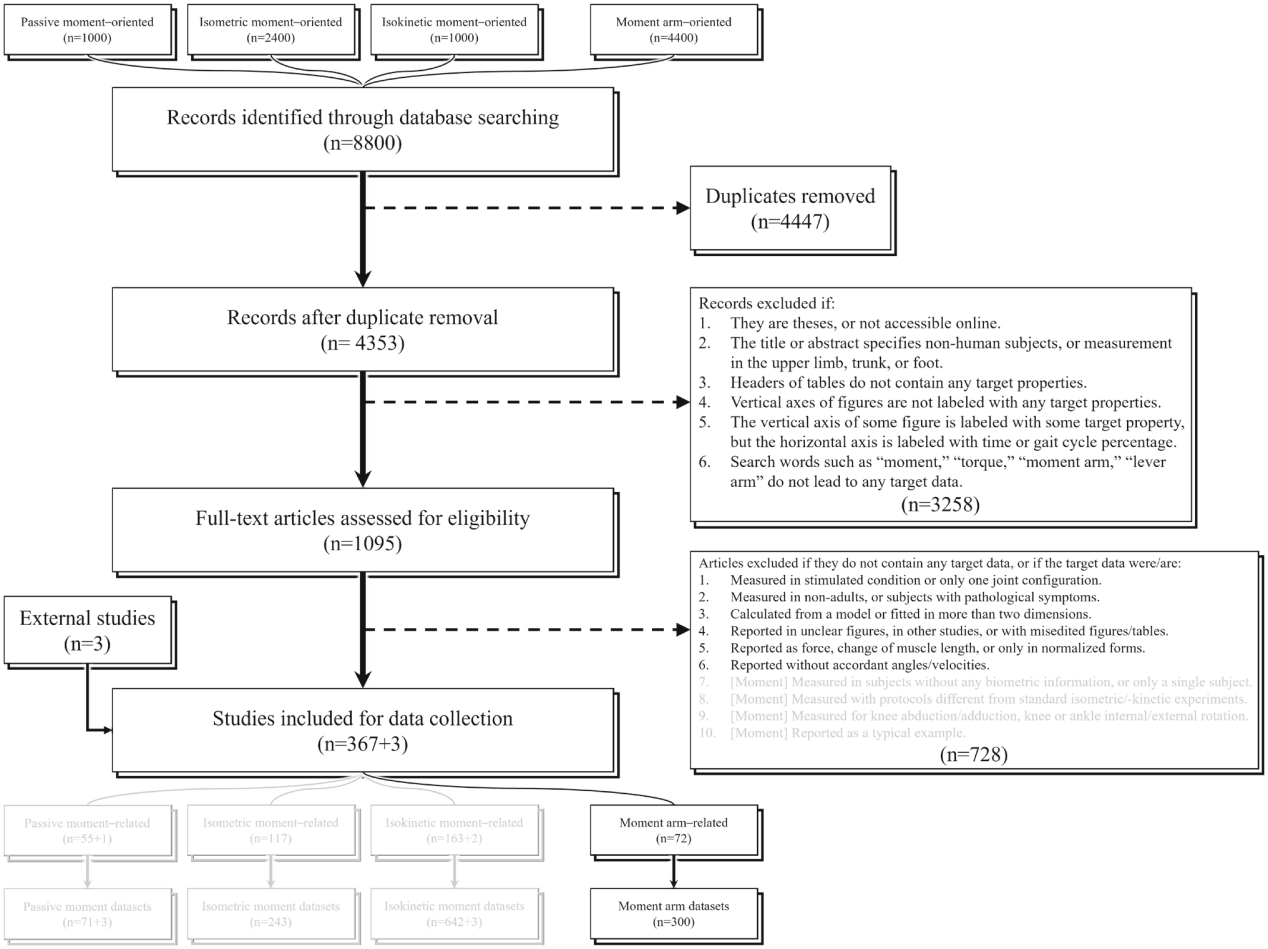
Workflow of study selection. Moment-related datasets are not presented in this study, but records identified by searches intended for moment data were examined for moment arm data

Other details of data collection are shown in Fig. 1 and can also be found in Supplementary Information. Note that we also initiated 4400 searches oriented toward joint moment for another study, but the search results were examined altogether. This serves as a compensation for the potential overfitting of the keywords from Table 1, in case some records are missed in the searches intended for moment arm data.

In data collection, if a study presents its data in graphs rather than in numbers, we use Graph Grabber (Version 2.0.2, Quintessa Ltd.) for manual digitization. The data are categorized by attributes listed in Table 2, where primary and secondary DoFs denote the variables (joint angle in the respective DoF) in moment arm measurement for the convenience of data storage. A dataset is generally defined as a set of measurements with distinction in either its reference, subject information, measured muscle, primary DoF, or data type. A muscle may have multiple moment arms, and the measured moment arm is indicated by the primary DoF. Meanwhile, data type denotes the method of measurement:

1. Two-dimensional center of rotation (CoR)
2. Three-dimensional center of rotation (CoR3)
3. Tendon excursion (TE)
4. Kinetic balance (KB).

**Table 2:** Metadata for moment arm datasets

Reference	Authors (Year)
Subject info	Sample size, sex, age height, weight, if in vivo
Muscle^a^	–
Data Type	CoR CoR3
	TE
	KB
Primary DoF^b^	Hip Extension/Flexion
	Hip Ab-/Adduction
	Hip Ex-/Internal Rotation
	Knee Extension/Flexion
	Ankle Plantar-/Dorsiflexion
	Ankle Eversion/Inversion
	Knee Ab-/Adduction
	Knee Ex-/Internal Rotation
	Ankle Ex-/Internal Rotation
Secondary DoF^b^	Knee Extension/Flexion
	Hip Extension/Flexion
	Ankle Plantar-/Dorsiflexion

^a^See Table 3

^b^The moment arm measured in a muscle is indicated by the primary DoF, which also determines the secondary DoF

When measured in vivo, a muscle could be in different states of activation, and for the potential need of comparative analysis, we labeled the data type with the plus sign as an approximate indication of muscle activation: not activated (no sign), activated up to 33% ($+$), 34–67% ($++$), and 67–100% ($+++$). In some studies, a muscle is treated as multiple strings or measured with the same method but different configurations (e.g., rotation center defined as different anatomical landmarks), and the results are recorded as different datasets with the configurations noted. For convenience, we provide a catalog with details of the datasets (see Supplementary Information).

In each dataset, there is at least one curve, which is comprised of measurements from at least two joint positions, and some datasets contain multiple curves, each comprised of multiple measurements. The data for each curve are stored as a 3-column matrix, with the first, second, and third column for the angles in the primary and secondary DoFs, and the moment arms. The sign rule for angle and moment arm in each DoF is based on the ISB recommendations [47, 48] except for knee flexion/extension, which is reversed so that moment arms for anti-gravity motions share the negative sign. If the angle in the secondary DoF is not specified in a study, we label it as NaN.

Since the data are mostly two dimensional and the measured joint positions are different across datasets, typical methods of meta-analysis no longer apply, and we did not proceed to combine the results. Also, considering the heterogeneity between studies, we refrain from making conclusive statements for any moment arm on the representative magnitude. For biomechanical analysis, readers are encouraged to select datasets with characteristics of subjects and means of measurement conforming to their object of study.

## Results

As shown in Fig. 1, a total of 72 studies were identified from 4353 records searched in the database, and they yielded 300 moment arm datasets. Table 3 shows the distribution of datasets. See “Appendix A” for a summary of collected studies and datasets.

**Table 3:** Distribution of datasets for moment arms in each lower limb muscle

Muscle	Number of Datasets^a^								
	Hip			Knee			Ankle		
	S	F	T	S	F	T	S	F	T
Achilles tendon							38	3	1
Soleus							1	–	–
Gastrocnemii				1	–	–	–	–	–
Gastrocnemius med				5	–	–	2	3	–
Gastrocnemius lat				5	–	–	1	3	–
Patellar tendon				40	–	–			
Quadriceps tendon				4	–	–			
Rectus femoris	1	–	–	7	–	–			
Vastus medialis				4	–	–			
Vastus lateralis				4	–	–			
Vastus intermedius				3	–	–			
Gluteus maximus	4	4	6	*4*					
Gluteus medius	3	–	6						
Gluteus minimus	–	–	–						
Biceps femoris				6	–	–			
Short head				1	–	1			
Long head	1	–	–	4	–	1			
Semimembranosus	1	–	–	8	–	1			
Semitendinosus	1	–	–	9	–	1			
Iliopsoas	–	–	1						
Psoas major	1	–	–						
Iliacus	–	–	–						
Tibialis posterior							5	7	1
Adductor lon./bre./mag	–	–	–						
Flexor hallucis longus							4	3	1
Flexor digitorum longus							2	3	1
Peroneus longus							3	3	1
Peroneus brevis							3	3	1
Tibialis anterior							16	8	1
Extensor hallucis longus							2	2	1
Extensor digitorum longus							2	2	1
Pectineus	–	–	–						
Piriformis	1	1	1						
Gracilis	–	–	–	7	–	1			
Tensor fasciae latae	2	2	–	*3*					
Sartorius	–	–	–	5	–	1			
Obturator internus	1	1	2						
Obturator externus	2	–	1						
Popliteus				1	–	1			
Gemelli	–	–	–						
Quadratus femoris	2	–	1						
Plantaris				–	–	–	–	–	–

^a^S: sagittal motion (hip or knee extension/flexion, ankle plantar-/dorsiflexion). F: frontal motion (hip or knee ab-/adduction, ankle eversion/inversion). T: transverse motion (ex-/internal rotation). The dash indicates that the muscle potentially actuates this DoF but no study has reported the accordant moment arm

**Fig. 2:**
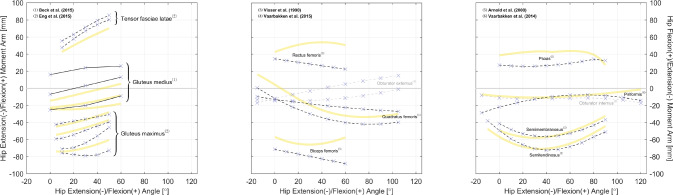
Relations of hip extension/flexion moment arms. Left: the gluteus maximus, gluteus medius, and tensor fasciae latae. Center and Right: the rectus femoris and other gluteal muscles with available data. All measurements were performed on cadaveric specimens of mixed/unknown sex (purple cross mark). The solid and dashed line styles denote, respectively, the CoR and TE methods. The yellow curves are model-derived moment arms from [49]: the glutei maximus and medius are both modeled as three muscle paths, and the obturator muscles are not modeled

**Fig. 3:**
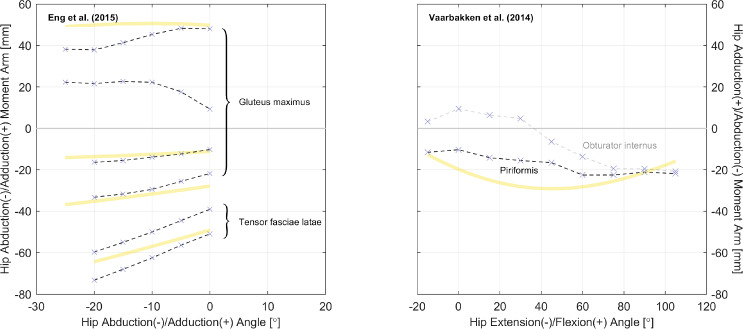
Relations of hip ab-/adduction moment arms. Left: relations with hip adduction angle. Right: relations with hip flexion angle. All measurements were performed on cadaveric specimens of mixed/unknown sex (purple cross mark) with the TE method (dashed line style). The yellow curves are model-derived moment arms from [49]: the gluteus maximus is modeled as three muscle paths, and the obturator internus is not modeled

**Fig. 4:**
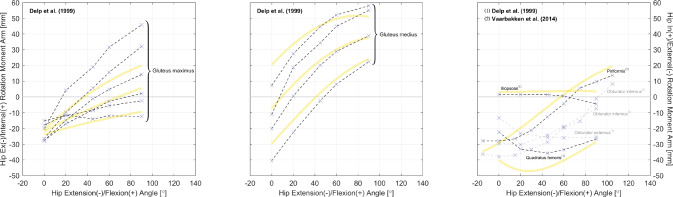
Relations of hip ex-/internal moment arms. Left and Center: the glutei maximus and medius. Right: other gluteal muscles with available data. All measurements were performed on cadaveric specimens of mixed/unknown sex (purple cross mark) using the TE method (dashed line style). The yellow curves are model-derived moment arms from [49]: the glutei maximus and medius are both modeled as three muscle paths, and the obturator muscles are not modeled

**Fig. 5:**
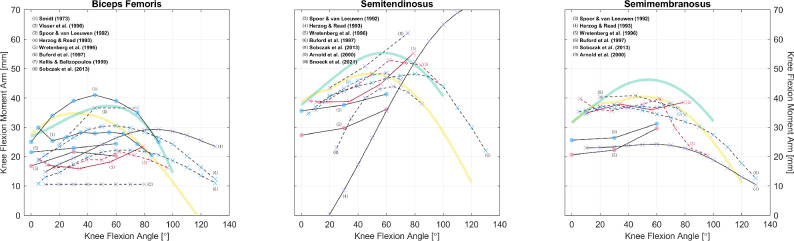
Relations of knee flexion moment arms of the hamstrings. Measurements were performed on healthy subjects (circle) or cadaveric specimens (cross mark). The scatter colors of red, blue, and purple denote, respectively, the sex of male, female, and mixed/unknown. The solid and dashed line styles denote, respectively, the CoR and TE methods; the different line and text colors are merely for convenience of distinction. The yellow and green curves are model-derived moment arms, respectively, from [49, 50]: outputs of the long head are plotted in representation of the biceps femoris

**Fig. 6:**
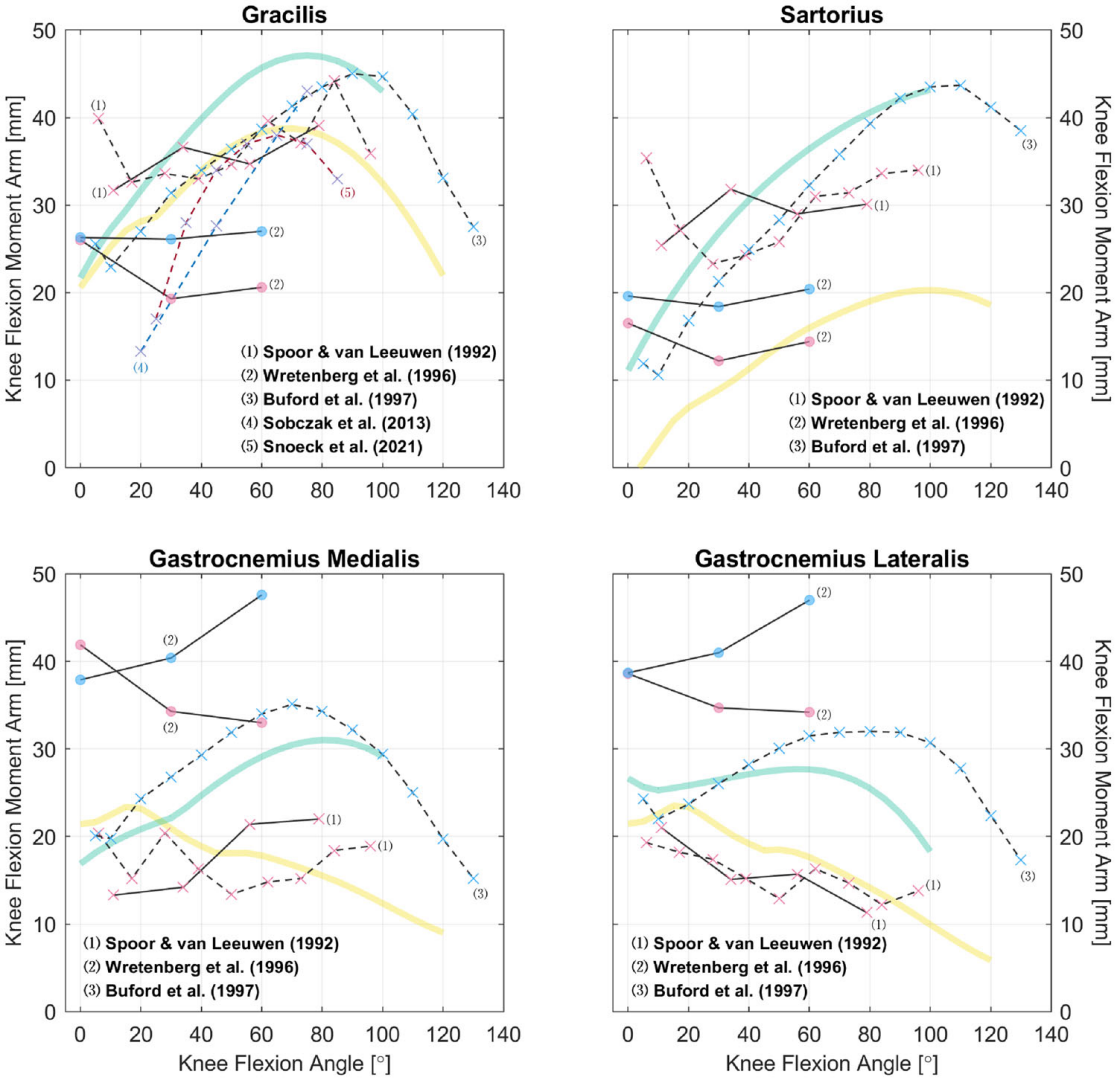
Relations of knee flexion moment arms of other knee flexors. Measurements were performed on healthy subjects (circle) or cadaveric specimens (cross mark). The scatter colors of red, blue, and purple denote, respectively, the sex of male, female, and mixed/unknown. The solid and dashed line styles denote, respectively, the CoR and TE methods; the different line and text colors are merely for convenience of distinction. The yellow and green curves are model-derived moment arms, respectively, from [49, 50]

**Fig. 7:**
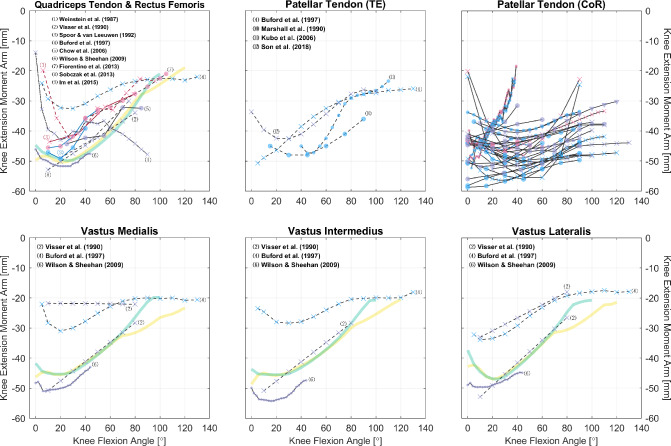
Relations of knee extension moment arms of the quadriceps and their tendons. Measurements were performed on healthy subjects (circle) or cadaveric specimens (cross mark). The scatter colors of red, blue, and purple denote, respectively, the sex of male, female, and mixed/unknown. The solid, dashed, and dotted line styles denote, respectively, the CoR, TE, and KB methods; the different line and text colors are merely for convenience of distinction. The yellow and green curves are model-derived moment arms, respectively, from [49, 50]. See “Appendix A” for detailed references for the patellar tendon

**Fig. 8:**
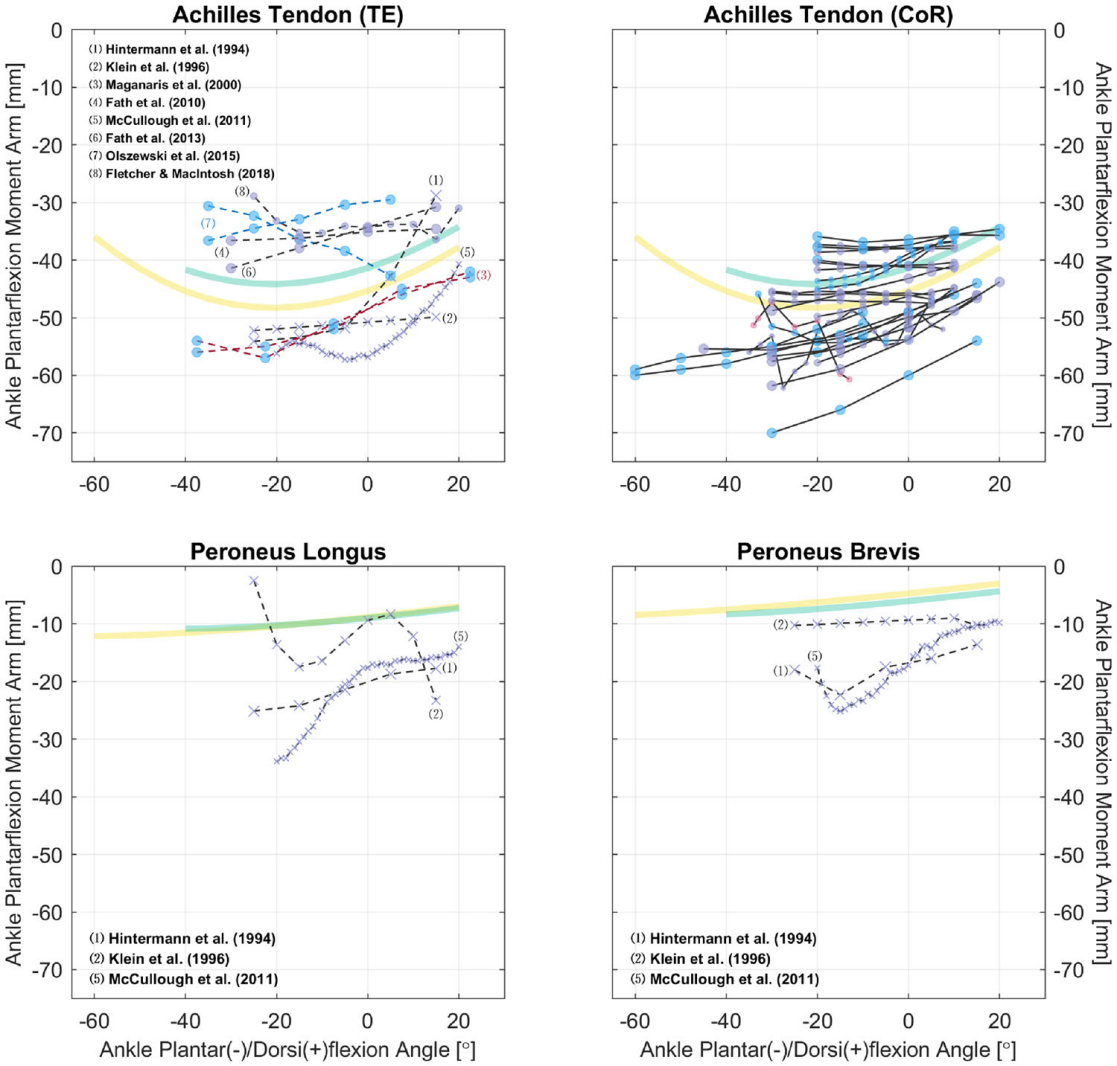
Relations of ankle plantar-/dorsiflexion moment arms of the Achilles tendon and peroneal muscles. Measurements were performed on healthy subjects (circle) or cadaveric specimens (cross mark). The scatter colors of red, blue, and purple denote, respectively, the sex of male, female, and mixed/unknown. The solid and dashed line styles denote, respectively, the CoR and TE methods; the different line and text colors are merely for convenience of distinction. The yellow and green curves are model-derived moment arms, respectively, from [49, 50]: outputs of the soleus are plotted in representation of the Achilles tendon. See “Appendix A” for detailed references for the Achilles tendon

**Fig. 9:**
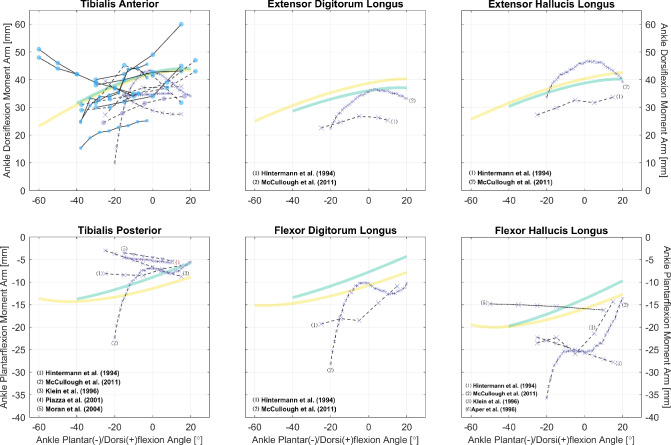
Relations of ankle plantar-/dorsiflexion moment arms of other leg muscles. Measurements were performed on healthy subjects (circle) or cadaveric specimens (cross mark). The scatter colors of blue and purple denote, respectively, the sex of male and mixed/unknown. The solid, dashed, and dotted line styles denote, respectively, the CoR, TE, and KB methods; the different line and text colors are merely for convenience of distinction. The yellow and green curves are model-derived moment arms, respectively, from [49, 50]. See “Appendix A” for detailed references for the tibialis anterior

**Fig. 10:**
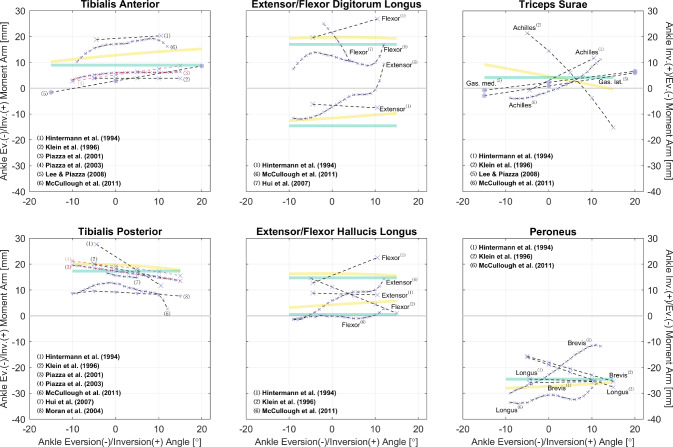
Relations of ankle eversion/inversion moment arms. All measurements were performed on healthy subjects of mixed sex (purple circle) or cadaveric specimens of mixed/unknown sex (purple cross mark) using the TE method (dashed line style); the different line and notation colors are merely for convenience of distinction. The yellow and green curves are model-derived moment arms, respectively, from [49, 50]: curves of the flexor digitorum/hallucis longus are above those of the extensors, and outputs of the peroneus longus are plotted in representation of the peroneus

The gathered datasets are mostly visualized in Figs. 2, 3, 4, 5, 6, 7, 8, 9 and 10. The sex of the subject group is indicated by the color of the scatters: red ($>80$% female), blue ($>80$% male), purple (mixed or unknown). The circle shape indicates in vivo measurements on young subjects, and the cross mark is for measurements from cadaveric specimens. The method of measurement is denoted by the line style: solid (CoR), dashed (TE), dash-dotted (KB). Only two datasets were measured using on the KB method [12, 13], whereas 189 were from the TE method. The CoR method contributed 109 datasets, of which 24 were measured based on a 3D center. Also, to demonstrate how these datasets can be of value to model calibration and validation, we also plot moment arm curves from two widely used musculoskeletal models [49, 50].

## Discussion

The goal of this study is to gather moment arm datasets and provide references for biomechanical analysis. Such information is especially critical for estimating muscle forces or activations in a given task. In this process, joint moments estimated from kinematic measurements must be distributed to each muscle and transformed into force or activation, both of which are dependent on moment arm. Critically, the accuracy of a musculoskeletal simulation, as well as the reliability of the drawn conclusions, is capped by the quality of moment arm data used for model calibration.

Although we launched a large-scale record search in the database, only a limited number of related studies were identified. This is not entirely surprising, since moment arm is largely an anatomical property, whose measurement is much more difficult than that of kinetic properties such as joint moment or external force. The two primary methods of moment arm measurement require either cadaveric specimens or medical imaging (e.g., MRI, X-ray, and ultrasound), both of which can be expensive and time-consuming.

As shown in Table 3, most datasets are for moment arms in the sagittal plane, and this matches our finding in gathered joint moment datasets [51]. In daily locomotion such as walking and running, the dynamics in the sagittal plane are more dominant compared with in the frontal and transverse planes [52, 53]. It is also typical to assume that the knee mainly functions in the sagittal plane, while the ankle does not rotate much. Hence, research interests tend to be focused on the sagittal plane. However, the ab-/adduction and ex-/internal rotation of the hip are too evident to ignore for accurate dynamic analysis, yet there are only three studies reporting the moment arm relations in a few related muscles (Figs. 3 and 4). For simulations investigating the dynamics in non-sagittal planes, it can be difficult to calibrate a musculoskeletal model based on existing datasets, and cautions should be given if conclusions are drawn without specifying the source of calibration data.

Even in the sagittal plane, many muscles lack sufficient data to reveal the characteristics of their moment arm–joint angle relations. In the hip, there are only six studies with relevant data: none is reported for the gluteus minimus, adductor longus/brevis/magnus, pectineus, or gemelli, while for the other hip muscles, each has measurements from only one study, with no additional studies available for comparison (Fig. 2). Moreover, most ankle muscles lack moment arm data even in the sagittal plane (Figs. 8 and 9). Similarly, musculoskeletal simulations may be questionable if they are designed to estimate the activities of these muscles.

Meanwhile, major muscle groups such as the quadriceps femoris, hamstrings, and triceps surae are frequently studied with many moment arm measurements. Nevertheless, it is important to note that muscle forces (or activations) are correlated throughout the lower limb: Overestimating the force of a muscle will certainly result in the agonist forces being underestimated and the antagonist forces being overestimated. This means that if too many muscles are not accurately calibrated, even the results of accurately calibrated muscles will likely suffer from error.

Another noteworthy point is that, although the quadriceps femoris, the biceps femoris, and the triceps surae each has its common insertion tendon, some studies report the separate moment arms of individual agonists [54–57]. This can be confusing because if the muscle line of action is set as the direction of the tendon, then according to the CoR method, muscles with a common tendon share the line of action and should have the same moment arm. However, based on the TE method, or the CoR method with differently defined lines of action, muscles with a common insertion may still have different length variations. For example, to measure excursion, Buford et al. sutured four cables for each of the quadriceps, and the insertions were selected as “the mid point of each muscle insertion in the quadriceps tendon” [54]. This way, the insertions become independent, and tendon excursion can be different as the joint rotates. Similarly, for each of the quadriceps, Wilson and Sheehan determined the lines of action as the direction of the muscles [56], and the measurement using the CoR method naturally differs.

To answer the question of which method is more accurate, it becomes critical to revisit the definition of moment arm as well as tendon anatomy. Essentially, it is implied in the CoR method that if muscles share a common tendon, force is uniformly distributed across the tendon, so the line of action remains unchanged regardless of which muscles are activated. On the other hand, the TE method assumes an independent line of action for each muscle even if they merge into one tendon. Anatomical experiments seem to support the latter. Grob et al. show that the quadriceps tendon is consisted of three layers, and the components of the second layer (the medial vastus medialis and the vastus lateralis) have different orientations [58]. Mahan et al. show that the Achilles tendon is a confluence of the gastrocnemius and the soleus, and it can be unbraided into three sub-tendons, and each inserts into their own calcaneal facets [59]. However, despite the anatomical distinction, the sub-tendons are intertwined with transverse interaction, so the force of a muscle might not be entirely transmitted via its own sub-tendon.

In theory, the method of kinetic balance should be the most accurate, where moment arm is calculated from the force measured on the muscle (or its substitute cable) to maintain a constant joint moment. The main advantage of this method is based on the kinetic definition of moment arm (Eq. 1). Regardless of how muscle force distributes in the tendon, it is directly matched with joint moment: either in vivo or in silico, the muscle has to generate the same amount of force to exert the same joint moment. In other words, this kinetic-based method is unaffected by tendon anatomy, and neither is there the need to consider muscle architecture, tendon curvature, or center of rotation, which are major concerns in the CoR method. A major disadvantage is the inconvenience of measurement, as it requires cadaveric specimens, force transducers, and a special apparatus to apply constant loads. This is likely the main reason why the KB method is not commonly seen in literature: In the studies we collected, only two designed experiments are based on the concept of kinetic balance [12, 13]. One other possibility is the lack of keywords specifically related to the KB method (e.g., “balance”), which we avoided so as not to *pollute* the search results by irrelevant topics such as balance control. However, even for the KB method, the center of rotation needs to be mentioned when explaining the mechanism, so related papers should be covered by our current keyword of “rotation,” though perhaps with a lower search prominence.

In addition to concerns regarding common tendons, there are pros and cons for both the CoR and TE methods. Generally, the TE method is similar to the KB method, except that it is more convenient. Only length is measured and the experiment can be performed without applying a constant joint load. Most importantly, there is no need to identify the rotation center. This perhaps explains a recent rise of studies performing the TE method in vivo with hand-held ultrasound [31, 36, 60, 61]. A major limitation of this method is that, the tendon may store elastic energy or undergo friction during joint rotation, violating the principle of virtual work, hence inducing errors in the measurements. Moreover, moment arm is the derivative of of length change with respect to joint angle, so the error in length measurement is easily magnified in derivation. If moment arm is calculated as the quotient of length difference and angle difference, it is typical to see moment arm that zig-zags as joint angle changes [46]. If the length data are fitted and moment arm is taken as the derivative of the fitting formula, then its relation with joint angle heavily depends on the choice of formula; e.g., it is linear if the fitting formula is quadratic [55, 57], or quadratic if the formula is cubic [34, 62, 63]. On the other hand, the CoR method directly measures some distance as moment arm, so it is free of this particular problem. However, whether this distance is an appropriate representation of moment arm depends on if the path of the muscle–tendon unit is straight, if the center of rotation is correctly identified, and if the muscle line of action is correctly identified. Due to these restrictions, the CoR method is mostly performed on muscles with large tendons, such as the quadriceps and triceps surae. Nonetheless, the identification of the center of rotation remains a problem for this method. For this, either the Reuleaux method is employed, or some anatomical landmark is assumed as the center. The latter is prone to error [41, 64], and if the center of rotation is moving rather than fixed, the Reuleaux method must be frequently repeated to ensure accuracy [9].

Critically, the center of rotation is often estimated in 2D, e.g., the sagittal plane, and the accordant 2D moment arm is in fact a projection of the true 3D moment arm onto the respective plane. In essence, the fundamental motions of the joint, such as hip abduction, knee flexion, or ankle inversion, are not completely confined to the sagittal, frontal, or transverse planes, hence their rotational axes are not strictly perpendicular to these planes. For example, knee flexion, as the motion observed in vivo, is mainly rotation in the sagittal plane, but accompanied with some level of rotation in both frontal and transverse planes. A good example is found in [65], which measured knee moment arms in both sagittal and frontal planes; e.g., the sagittal and frontal moment arms of the patellar tendon are approximately $-45$ and $-5$ mm. This does not necessarily mean that the moment actuating knee extension is nine times than the moment actuating knee abduction, but rather knee extension/flexion itself may contain partial rotation in the frontal plane, and the force exerted by the quadriceps could all be transformed into knee extension moment; in this case, the true knee extension moment arm of the patellar tendon is $\sqrt{45^2+5^2}=45.3$ mm. This means that when the moment arm data for calibration are obtained using the 2D CoR method, it is important to check if the rotation axis in measurement matches with that in the musculoskeletal model. If the model rotation axis is not perpendicular to the reference planes, then data obtained via the 2D CoR method will underestimate the model moment arm. Similarly, moment arm measured using the other methods is based on a *natural* rotation axis, so it is important to configure the model with a similar axis. Otherwise if a perpendicular axis is configured, the model moment arm might be overestimated.

So far, we have only utilized the collected studies to cover important moment arm–related topics, without combining the results shown in Figs. 2, 3, 4, 5, 6, 7, 8, 9 and 10 using meta-analysis for extensive discussion. This is indeed a limitation of our work, and it would be helpful if we could derive some aggregated moment arm–joint angle relations. However, we try not to mislead readers with such results, since the collected studies are heterogeneous in terms of subject demographics and anthropometrics as well as measurement type (in vivo or cadaveric; muscle activated or not) and method. That said, our data collection still offers a general and direct impression of the pattern and magnitude of many moment arms, which can be useful in biomechanical analysis. For most muscles, the available datasets are too scarce to choose in alignment with specific study objectives. Whereas for major muscles such as the hamstrings (Fig. 5), the quadriceps (Fig. 7, top), the triceps surae (Fig. 8, top), and the tibialis anterior (Fig. 9, top left), readers may select from datasets to match their study objectives (see Supplementary Information). Suppose one seeks to compare the computed knee contact force between toe-in/out gaits in young female, then it becomes necessary to update models such as [49] and [50]—whose knee moment arms are validated against cadaveric measurements—with in vivo data from young female; or at least subjects with a similar age. Likewise, based on the patient’s sex, age, height, and weight, surgeons may refer to the appropriate datasets to determine the optimal surgical site, e.g., the insertion point in Achilles tendon repair or the rotation center in knee replacement, for restoring natural moment arm relations.

Finally, we expand on the topic of musculoskeletal modeling. An ideal calibration of a musculoskeletal model requires the high-dimensional relations between all moment arms of a muscle and all its related joint angles [66]. This was one other motive of our study, but most of the gathered datasets are about the relation between moment arm and angle in the same plane; except for [16, 62], and [67]. For accurate musculoskeletal modeling, it is necessary to obtain moment arm data for as many actuating DoFs in as many muscles, and Table 3 should serve as a good reference to experiment design for gap-filling measurements. Currently, several approaches may be taken in compensation for the lack of experimental data. If 3D MRI reconstruction is available, moment arm can be estimated using the CoR method. For convenience, only one position is needed, and the rotation axes for measurement can be matched with those configured in the musculoskeletal model. This way, a rough estimation of the moment arm magnitude can be obtained. As a less preferable alternative, one could model an initial muscle path based on the anatomical origin and insertion points in the skeletal model, and take the distance between muscle path and rotation axis as moment arm. With the estimated magnitude, one must then assume a certain relation for the variation of moment arm in different joint positions. This could be assumed as constant, or similar to that of the agonists. For example, the glutei maximus and medius tend to function as hip external rotators when the hip is extended or slightly flexed, and they gradually become internal rotators as in highly flexed hip positions [68]. So a similar pattern can be assigned to the gluteus minimus. In contrast, for hip muscles that are not typically known as rotators, their rotation moment arms can be treated as a small constant. As a last step, with the estimated magnitude and assumed relations, moment arms may be generated for various joint positions and serve as calibration data for automated muscle path calibration [66].

## Conclusion

A total of 300 moment arm datasets were collected from literature to illustrate muscle moment arm–joint angle relations in the hip, knee, and ankle. The overall pattern and magnitude are presented for six actuated DoFs. The findings should provide insight into musculoskeletal mechanics and improve the design of future experiments for moment arm measurement.

## Supplementary Information

Below is the link to the electronic supplementary material.

Supplementary file 1 (zip 4295 KB)

## Data Availability

Files associated with this article, including the search log and metadata catalog (.xlsx), datasets (.mat), and visualization scripts (.m), can be found in Supplementary Information. They are also available at https://doi.org/10.6084/m9.figshare.26018563, with potential corrections and updates of the datasets in the future.

## Appendix A: Summary of studies and datasets

**Table:**

Dataset^a^	Study	Method(s)
Gluteus maximus	Eng et al. (2015) [15]	TE
Gluteus medius	Beck et al. (2015) [69]	CoR
Tensor fasciae latae	Eng et al. (2015) [15]	TE
Psoas	Arnold et al. (2000) [70]	TE
Piriformis	Vaarbakken et al. (2014) [62]	TE
Obturator internus	Vaarbakken et al. (2014) [62]	TE
Obturator externus	Vaarbakken et al. (2015) [63]	TE
Quadratus femoris	Vaarbakken et al. (2015) [63]	TE
Rectus femoris	Visser et al. (1990) [57]	TE
Biceps femoris (long)	Visser et al. (1990) [57]	TE
Semitendinosus	Arnold et al. (2000) [70]	TE
Semimembranosus	Arnold et al. (2000) [70]	TE
Gluteus maximus	Eng et al. (2015) [15]	TE
Tensor fasciae latae	Eng et al. (2015) [15]	TE
Piriformis	Vaarbakken et al. (2014) [62]	TE
Obturator internus	Vaarbakken et al. (2014) [62]	TE
Gluteus maximus	Delp et al. (1999) [68]	TE
Gluteus medius	Delp et al. (1999) [68]	TE
Iliopsoas	Delp et al. (1999) [68]	TE
Piriformis	Vaarbakken et al. (2014) [62]	TE
Obturator internus	Vaarbakken et al. (2014) [62]	TE
	Delp et al. (1999) [68]	TE
Obturator externus	Delp et al. (1999) [68]	TE
Quadratus femoris	Delp et al. (1999) [68]	TE
Biceps femoris	Smidt (1973) [10]	CoR
	Spoor and van Leeuwen (1992) [46]	CoR, TE
	Buford et al. (1997) [54]	TE
	Sobczak et al. (2013) [71]	TE
	Visser et al. (1990) [57]	TE
	Wretenberg et al. (1996) [65]	CoR
	Herzog and Read (1993) [7]	CoR
	Kellis and Baltzopoulos (1999) [72]	CoR
Semitendinosus	Spoor and van Leeuwen (1992) [46]	CoR, TE
	Arnold et al. (2000) [70]	TE
	Buford et al. (1997) [54]	TE
	Sobczak et al. (2013) [71]	TE
	Wretenberg et al. (1996) [65]	CoR
	Herzog and Read (1993) [7]	CoR
	Snoeck et al. (2021) [73]	TE
Semimembranosus	Spoor and van Leeuwen (1992) [46]	CoR, TE
	Arnold et al. (2000) [70]	TE
	Buford et al. (1997) [54]	TE
	Sobczak et al. (2013) [71]	TE
	Wretenberg et al. (1996) [65]	CoR
	Herzog and Read (1993) [7]	CoR

**Table:**

Gracilis	Spoor and van Leeuwen (1992) [46]	CoR, TE
	Buford et al. (1997) [54]	TE
	Sobczak et al. (2013) [71]	TE
	Wretenberg et al. (1996) [65]	CoR
	Snoeck et al. (2021) [73]	TE
Sartorius	Spoor and van Leeuwen (1992) [46]	CoR, TE
	Buford et al. (1997) [54]	TE
	Wretenberg et al. (1996) [65]	CoR
Gastrocnemius	Spoor and van Leeuwen (1992) [46]	CoR, TE
	Buford et al. (1997) [54]	TE
	Visser et al. (1990) [57]	TE
	Wretenberg et al. (1996) [65]	CoR
Patellar tendon	Smidt (1973) [10]	CoR
	Buford et al. (1997) [54]	TE
	Wilson and Sheehan (2009) [56]	CoR3
	Wretenberg et al. (1996) [65]	CoR
	Herzog and Read (1993) [7]	CoR
	Kellis and Baltzopoulos (1999) [72]	CoR
	Westphal et al. (2013) [74]	CoR3
	Tsaopoulos et al. (2009) [64]	CoR
	Tsaopoulos et al. (2007) [75]	CoR
	Im et al. (2015) [44]	CoR
	Dandridge et al. (2022) [43]	CoR3
	Sheehan (2007) [45]	CoR, CoR3
	Reeves et al. (2004) [76]	CoR
	Finni et al. (2001) [77]	CoR
	Krevolin et al. (2004) [20]	CoR3
	van Eijden et al. (1987) [78]	CoR
	Marshall et al. (1990) [79]	TE
	Ward et al. (2012) [80]	CoR, CoR3
	Baltzopoulos (1995) [81]	CoR
	Kubo et al. (2006) [82]	TE
	Gray et al. (2021) [19]	CoR
	Ward et al. (2005) [83]	CoR
	Kefala et al. (2022) [84]	CoR3
	Hosseinzadeh et al. (2020) [85]	CoR
	Son et al. (2018) [86]	TE
	Nakamura et al. (1985) [87]	CoR
Quadriceps tendon	Im et al. (2015) [44]	CoR
	Weinstein et al. (1987) [13]	KB
	Chow et al. (2006) [88]	CoR

**Table:**

Rectus femoris	Spoor and van Leeuwen (1992) [46]	CoR, TE
	Buford et al. (1997) [54]	TE
	Sobczak et al. (2013) [71]	TE
	Visser et al. (1990) [57]	TE
	Wilson and Sheehan (2009) [56]	CoR3
	Fiorentino et al. (2013) [89]	TE
Vastus med./int./lat.	Buford et al. (1997) [54]	TE
	Visser et al. (1990) [57]	TE
	Wilson and Sheehan (2009) [56]	CoR3
Gluteus maximus	Eng et al. (2015) [15]	TE
Tensor fasciae latae	Spoor and van Leeuwen (1992) [46]	TE
	Sobczak et al. (2013) [71]	TE
	Eng et al. (2015) [15]	TE
Popliteus	Buford et al. (1997) [54]	TE
Triceps Surae	Klein et al. (1996) [34]	TE
	Hintermann et al. (1994) [16]	TE
	Maganaris (2003) [90]	TE
	McCullough et al. (2011) [17]	TE
	Hashizume et al. (2012) [6]	CoR, CoR3
	Clarke et al. (2015) [29]	CoR3
	Rugg et al. (1990) [9]	CoR
	Maganaris et al. (2000) [8]	CoR, TE
	Fletcher and MacIntosh (2018) [32]	TE
	Sheehan (2012) [40]	CoR3
	Wade et al. (2019) [41]	CoR, CoR3
	Franz et al. (2019) [91]	CoR
	Manal et al. (2013) [36]	CoR
	Olszewski et al. (2015) [38]	TE
	Fath et al. (2010) [31]	CoR, TE
	Wolfram et al. (2018) [67]	CoR
	Csapo et al. (2013) [92]	CoR
	Fath et al. (2013) [60]	CoR, TE
	Yeh et al. (2021) [93]	CoR
	Obst et al. (2017) [37]	CoR3
	Wang et al. (2021) [94]	TE
Peroneus longus/brevis	Klein et al. (1996) [34]	TE
	Hintermann et al. (1994) [16]	TE
	McCullough et al. (2011) [17]	TE
Tibialis anterior	Klein et al. (1996) [34]	TE
	Hintermann et al. (1994) [16]	TE
	McCullough et al. (2011) [17]	TE
	Piazza et al. (2001) [95]	TE
	Sasaki et al. (2014) [96]	TE
	Rugg et al. (1990) [9]	CoR
	Miller et al. (2015) [61]	CoR, TE
	Maganaris (2000) [97]	CoR, TE
	Ito et al. (2000) [98]	CoR

**Table:**

Extensor digitorum longus	Hintermann et al. (1994) [16]	TE
	McCullough et al. (2011) [17]	TE
Extensor hallucis longus	Hintermann et al. (1994) [16]	TE
	McCullough et al. (2011) [17]	TE
Tibialis posterior	Klein et al. (1996) [34]	TE
	Hintermann et al. (1994) [16]	TE
	McCullough et al. (2011) [17]	TE
	Piazza et al. (2001) [95]	TE
	Moran et al. (2004) [99]	TE
Flexor digitorum longus	Hintermann et al. (1994) [16]	TE
	McCullough et al. (2011) [17]	TE
Flexor hallucis longus	Klein et al. (1996) [34]	TE
	Hintermann et al. (1994) [16]	TE
	McCullough et al. (2011) [17]	TE
	Aper et al. (1996) [12]	KB
Tibialis anterior	Klein et al. (1996) [34]	TE
	Hintermann et al. (1994) [16]	TE
	Lee and Piazza (2008) [55]	TE
	McCullough et al. (2011) [17]	TE
	Piazza et al. (2001) [95]	TE
	Piazza et al. (2003) [100]	TE
Extensor digitorum longus	Hintermann et al. (1994) [16]	TE
	McCullough et al. (2011) [17]	TE
Flexor digitorum longus	Hintermann et al. (1994) [16]	TE
	McCullough et al. (2011) [17]	TE
	Hui et al. (2007) [101]	TE
Triceps Surae	Klein et al. (1996) [34]	TE
	Hintermann et al. (1994) [16]	TE
	Lee and Piazza (2008) [55]	TE
	McCullough et al. (2011) [17]	TE
Tibialis posterior	Klein et al. (1996) [34]	TE
	Hintermann et al. (1994) [16]	TE
	McCullough et al. (2011) [17]	TE
	Piazza et al. (2001) [95]	TE
	Hui et al. (2007) [101]	TE
	Piazza et al. (2003) [100]	TE
	Moran et al. (2004) [99]	TE
Extensor hallucis longus	Hintermann et al. (1994) [16]	TE
	McCullough et al. (2011) [17]	TE
Flexor hallucis longus	Klein et al. (1996) [34]	TE
	Hintermann et al. (1994) [16]	TE
	McCullough et al. (2011) [17]	TE
Peroneus longus/brevis	Klein et al. (1996) [34]	TE
	Hintermann et al. (1994) [16]	TE
	McCullough et al. (2011) [17]	TE
Biceps femoris	Buford et al. (2001) [102]	TE
Semitendinosus	Buford et al. (2001) [102]	TE

**Table:**

Semimembranosus	Buford et al. (2001) [102]	TE
Gracilis	Buford et al. (2001) [102]	TE
Sartorius	Buford et al. (2001) [102]	TE
Popliteus	Buford et al. (2001) [102]	TE
Tibialis anterior	McCullough et al. (2011) [17]	TE
Extensor digitorum longus	McCullough et al. (2011) [17]	TE
Flexor digitorum longus	McCullough et al. (2011) [17]	TE
Achilles tendon	McCullough et al. (2011) [17]	TE
Tibialis posterior	McCullough et al. (2011) [17]	TE
Extensor hallucis longus	McCullough et al. (2011) [17]	TE
Flexor hallucis longus	McCullough et al. (2011) [17]	TE
Peroneus longus/brevis	McCullough et al. (2011) [17]	TE
